# From the Field to the Screen: A Scoping Review of Video Analysis as a Tool for Understanding Thigh Muscle-Tendon Injuries in Football

**DOI:** 10.1097/JSM.0000000000001377

**Published:** 2025-06-13

**Authors:** Stefano Palermi, Filippo Vittadini, Carles Pedret, Marco Vecchiato, Francesco Della Villa, Alessandro Corsini, Aleksi Jokela, Lasse Lempainen

**Affiliations:** * UniCamillus Saint Camillus International University of Health Sciences, Rome, Italy;; † Venezia FC, Venezia, Italy;; ‡ Sports Medicine and Imaging Department, Clinica Diagonal, Barcelona, Spain;; § Sports and Exercise Medicine Division, Department of Medicine, University of Padova, Padova, Italy;; ¶ Education and Research Department, Isokinetic Medical Group, FIFA Medical Centre of Excellence, Bologna, Italy;; ║ Genoa CFC, Genoa, Italy;; ** Department of Surgery, Hospital Satasairaala, Pori, Finland;; †† Department of Physical Activity and Health, Paavo Nurmi Centre, University of Turku, Turku, Finland; and; ‡‡ FinnOrthopaedics, Hospital Pihlajalinna, Turku, Finland.

**Keywords:** muscle injuries, tendon injuries, video analysis, injury prevention, injury mechanisms, hamstrings

## Abstract

**Objective:**

Muscle-tendon injuries are critical setbacks in professional football, significantly affecting player availability and team performance. Understanding these injury mechanisms through video analysis is crucial for developing effective prevention and rehabilitation strategies that enhance player welfare and optimize performance. This review aimed to synthesize data from articles that used video analysis to explore mechanisms of thigh muscle-tendon injuries in football.

**Data source:**

A comprehensive literature search was conducted from 2010 to 2025 using a scoping review methodology. The quality of the included studies was assessed using the Quality Appraisal for Sports Injury Video Analysis Studies (QA-SIVAS) scale.

**Main results:**

The review of 10 studies identified that noncontact and indirect contact mechanisms are predominantly responsible for severe thigh muscle-tendon injuries in football. Common injury scenarios involved sprinting-induced strains and kicking actions, highlighting the significant role of eccentric loading and rapid biomechanical changes.

**Conclusions:**

Video analysis has emerged as a vital tool in sports medicine, providing deep insights into the complex mechanisms of thigh muscle-tendon injuries in football. The continuous improvement of analytical methods, including the adoption of advanced technologies such as artificial intelligence, is imperative for refining prevention and rehabilitation protocols.

## INTRODUCTION

Minimizing the impact of injuries is essential for the medical and performance teams in football, given the significant economic and competitive consequences associated with players' time away from the field.^1,2^ Muscle-tendon injuries, the most prevalent type of injury among elite male footballers,^3^ account for approximately one-third of the total time lost due to injuries.^4,5^ Notably, hamstring injuries have become increasingly common, now representing nearly one in every four reported injuries.^6,7^ Even if most muscle injuries result in relatively short layoff times (within 2 to 3 weeks), approximately 11% are severe, with absences exceeding 28 days.^8^ These severe injuries pose considerable treatment challenges and carry a high risk of recurrence.^9–11^

Muscle-tendon injuries affect a typical 25-player football squad significantly,^11^ with approximately 15 injuries per season leading to an average absence of 14.4 days and approximately 220 days of absence per team annually.^8^ Most (92%) of these injuries affect the four major muscle groups of the lower limb, especially of the thigh: hamstrings (37%), adductors (23%), quadriceps (rectus femoris) (19%), and calf muscle too (13%).^12–14^ Understanding the mechanisms of injury, which can be multifactorial involving biological processes, imaging, and athlete history, is crucial for an accurate rehabilitation pathway and a proper prognosis.^15^

Identifying the mechanisms behind these injuries, including both direct and indirect, as well as contact and non-contact, is a pivotal step in developing effective injury prevention strategies.^16,17^ Although athlete interviews provide extensive data, they often lack clarity in delineating injury moments.^18^ By contrast, video analysis, through detailed frame-by-frame slow motion and video stoppage, offers a more accurate depiction of the contributory factors to an injury.^19,20^ Despite not being the definitive method for analyzing the kinematics of injuries, video analysis is recognized as a valid tool for examining the context of injuries, including mechanisms and situational patterns—terms that describe the conditions under which injuries occur, influenced by factors such as ball possession and playing action.^21,22^ Video analysis has been effectively used to elucidate injury mechanisms and situational patterns across a spectrum of pathologies in various sports.^23–27^

Given the critical role of understanding injury mechanisms in preventing sports injuries, the importance of video analysis cannot be overstated. However, to date, its use is not widespread. Therefore, this study aimed to conduct a scoping review of the existing literature on thigh muscle-tendon injury video analysis studies in football to synthesize the current understanding of the injury mechanisms of these muscles.

## METHODS

**Figure 1.**
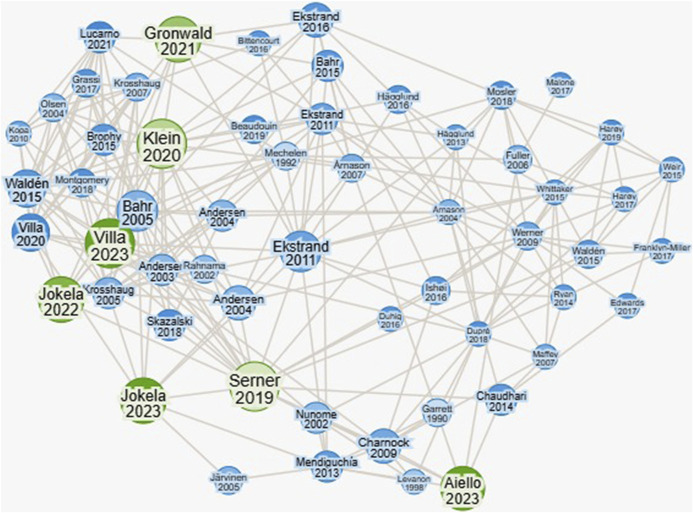
Graphical representation of the interconnections between video analysis studies on muscle-tendon injuries in athletes, created by Research Rabbit. Each node represents a study, with the node size indicating citation frequency and influence in the field. The included studies are highlighted in green, and the arrows represent the citation relationships, illustrating the flow of ideas and developments across studies.

We initiated our review with a preliminary search using ResearchRabbit (https://www.researchrabbit.ai/), an Artificial Intelligence (AI)–based search engine, to map the landscape of research interest in muscle-tendon injuries analyzed through video within football. This investigation, visualized in Figure 1, highlights body of research, setting the foundation for a comprehensive scoping review.

### Scoping Review Aims

Our scoping review aimed to explore the extent, range, and nature of research activities concerning video analysis of thigh muscle-tendon injuries in professional football, identifying emerging trends and gaps in the literature.

### Search Strategy

We conducted a comprehensive search using multiple databases, including PubMed, Scopus, Cochrane, Embase, SportDiscuss, and Google Scholar. The search used specific keywords related to “muscle injuries,” “tendon injuries,” “video analysis,” “football,” and “sports injuries,” without restricting the search to peer-reviewed articles alone. This allowed the inclusion of gray literature, conference papers, and thesis projects to ensure comprehensive coverage of the topic. The literature search was conducted from inception to February 2025 to capture all relevant studies.

### Inclusion and Exclusion Criteria

Our review included studies that use video analysis as a primary method for investigating thigh muscle-tendon injuries in professional football athletes. We considered studies focusing only on acute thigh muscle-tendon injuries. We excluded studies that did not use video analysis, focused on other sports disciplines, or investigated other sports injuries (eg, anterior cruciate ligament injuries or Achilles tendon ruptures). Articles not written in English were also excluded.

### Data Extraction

For each selected study, we extracted key information, including the study's objectives, the number of injuries analyzed, the muscle groups examined, and the main findings related to injury mechanisms and patterns.

### Quality Assessment

The methodological quality of the included studies was critically appraised using the Quality Appraisal for Sports Injury Video Analysis Studies (QA-SIVAS) scale, which comprises an 18-item checklist evaluating study design, data sources, methodology, and reporting.^28^ With its 18-item checklist, it offers a comprehensive framework for assessing the quality of video analysis studies in sports injury research, focusing on study design, data sources, conduct, reporting, and discussion.^28^

### Equity, Diversity, and Inclusion Statement

The author group is balanced, comprising junior, mid-career, and senior researchers from various disciplines; moreover, all members of the author group are from different countries. Our review focused on both male and female football players. The influence of gendered environments on injury is discussed.

## RESULTS

In the present review, we included 10 studies investigating video analysis techniques to analyze thigh muscle-tendon injuries in football^26,29–37^ (Table 1).

**TABLE 1.** Details of Included Studies

Study Citation	Design	Population	Injury Focus	Main Injury Mechanisms Identified	Data Source
Della Villa et al^50^	Cross-sectional observational study	103 cases	Lower-limb muscle injuries	Noncontact mechanisms	Video analysis, public online resources
Della Villa et al^49^	Cross-sectional observational study	129 cases	Hamstring injuries (male vs female)	Running, overstretching, and kicking, with notable gender differences	Video analysis, public online resources
Gronwald et al^20^	Cross-sectional observational study	52 cases	Hamstring injuries	Rapid movements with eccentric contractions	Video analysis, medical reports
Jokela et al^25^	Case series	14 cases	Hamstring injuries	Sprint, stretch, and mixed-type	Video analysis, MRI findings
Jokela et al^24^	Case series	20 cases	Rectus femoris injuries	Kicking	Video analysis, MRI findings
Klein et al^28^	Case series	80 cases	Thigh injuries	Sprinting, running, and lunging	Video analysis, public online resources
Serner et al^44^	Cross-sectional	17 cases	Adductor longus injuries	Noncontact incidents during rapid closed and open chain movements	Video analysis
Aiello et al^36^	Retrospective descriptive study	46 cases	Lower-limb muscle injuries	Deceleration, acceleration with ball control, or kicking + most injuries occurred >25 km/h, and above 80% of players' maximal sprinting capacity	Video analysis + GPS data (Catapult Vector S7)
Jokela et al^35^	Cross-sectional	20 cases	Adductor longus injuries	Closed kinetic chain actions, often while reaching with the uninjured leg	Video analysis, MRI findings
Vermeulen et al^37^	Prospective observational cohort study	63 cases	Hamstring injuries	Acceleration over short distances (<10 m). Pressing actions (46%) and indirect contact (53%) were common contributors	Video analysis, public online resources

Serner et al^33^ provided an in-depth analysis of the injury mechanisms associated with 17 cases of adductor longus injuries. Klein et al^31^ explored injury patterns across 81 thigh muscle injuries as part of an extensive video analysis endeavors,^32^ encompassing a broad array of football-related injuries. Similarly, Gronwald et al^32^ offered insights into the patterns of hamstring injuries among 52 professional male football players. Jokela et al^26^ introduced a novel approach by correlating magnetic resonance imaging (MRI) findings with video analysis outcomes, focusing on 14 high-grade hamstring injuries and 20 rectus femoris injuries^30^ The same authors (Jokela et al^35^), years later, conducted a systematic video analysis of 20 severe adductor longus injuries. Aiello et al^36^ introduced a novel approach by integrating Global Positioning System (GPS) data with video analysis to assess noncontact injuries. Vermeulen et al^37^ expanded on hamstring injury mechanisms by analyzing 63 sudden-onset injuries in professional football. Della Villa et al^29^ expanded the scope of video analysis, encompassing 103 muscle injuries, including injuries to the hamstring, calf, adductor, and quadriceps muscles. Finally, in a recent article, the same authors (Della Villa et al^34^) conducted a comparative video analysis of 129 hamstring injury mechanisms in 65 professional male and 64 female football players.

### Thigh

The analysis of thigh injuries, including all four major muscles, has been the subject of three pivotal studies^29,31,36^ that have shed light on the patterns and mechanisms behind acute injuries in professional football.

The study by Klein et al,^31^ provides a comprehensive analysis of acute injuries in professional men's football, focusing on describing typical injury patterns. Through a systematic analysis of video footage of moderate and severe acute match injuries from the two highest divisions in German male football over three seasons (2014-2017) (4.3% goalkeepers, 39.7% defenders, 41.4% midfielders, and 14.5% forwards), the study identifies nine typical injury patterns, including two specifics to thigh injuries: the “Sprinter's thigh injury” and the “Perturbation-and-strain thigh injury.” Half of the injuries occurred during game situations with the ball, with players in possession of the ball in 76.1% of the cases. Running (27.2%) and sprinting (23.2%) were the most common basic movement patterns of the injured player at the time of injury. Most injuries occurred during duels, mainly during tackles of the opponent. Thigh injuries (16 quadriceps, 40 hamstrings, and 24 adductors), predominantly resulting from noncontact situations (54.3% of thigh injuries were noncontact), comprise a significant portion of the study. These injuries were characterized by rapid force movements with high eccentric loads, especially evident in the “Sprinter's Thigh Injury” pattern, highlighting the significant role of eccentric loading during high-speed running or sprinting in injury causation.

Aiello et al^36^ introduced an innovative approach by integrating GPS data with video analysis to assess the inciting circumstances of noncontact injuries in elite football players. Their study analyzed 34 noncontact injuries among 46 male elite players from one Serie A football club over three seasons, identifying that most hamstring injuries (88%) occurred when players reached speeds greater than 25 km/h, often exceeding 80% of their maximal sprinting velocity. Furthermore, both quadriceps injuries occurred while players were kicking: in one case, the player was kicking the ball while walking, and in the other, the player was kicking the ball while running. Adductor injuries exhibited diverse mechanisms, including deceleration without the ball, acceleration while controlling the ball, and performing an instep kick. These findings suggest that limiting high-speed exposures, as sometimes proposed in injury prevention strategies, may not be advisable, as injuries frequently occur at peak running speeds.

The study conducted by Della Villa et al^29^ meticulously examines the causative factors behind severe muscle injuries in professional soccer. Focusing on injuries resulting in lay-off times exceeding 28 days, this research identified 121 severe lower-limb muscle injuries across three consecutive Italian Serie A seasons, with detailed video analysis being possible for 103 instances (comprising 4 goalkeepers, 45 defenders, 27 midfielders, and 27 forwards). The injuries predominantly affected the hamstrings (60%), followed by the calf (16%), adductors (15%), and quadriceps (9%) muscles. The average lay-off time for all injuries was 42.6 ± 20.1 days, with specific averages for hamstring (39.8 ± 20.6 days), calf (39.7 ± 3.9 days), adductor (54.2 ± 23.8 days), and quadriceps injuries (46.4 ± 24.8 days). More injuries occurred in offensive (n = 61, 59%) than in defensive situations (n = 42, 41%), with quadriceps injuries being particularly common in offensive plays (89%). The study revealed that a significant majority (83%) of the injuries were noncontact, underscoring the prevalence of indirect contact mechanisms. Four primary situational patterns accounted for 88% of the injuries: running/acceleration, closed kinetic chain (CKC) stretching, open kinetic chain (OKC) stretching, and kicking. These patterns highlight the variability in injury mechanisms across different muscle groups. Running/acceleration injuries were the most common (n = 35, 34%), representing the typical posterior kinetic chain injuries (hamstring and calf) pattern. These injuries occurred during high-speed running (n = 25), predominantly affecting the hamstrings (n = 24, 39% of all hamstring injuries). Overstretching-type injuries collectively accounted for two-fifths of the injuries (39%), with a similar distribution between CKC (n = 21, 20%) and OKC situations (n = 19, 18%). CKC injuries were apparent in the hamstrings (n = 12, 19%), calves (n = 7, 41%), and quadriceps muscles (n = 2, 22%). OKC stretching injuries affected the hamstrings (n = 15, 26%) and adductors (n = 4, 25%). Kicking injuries accounted for one in six injuries (n = 16, 16%), affecting the quadriceps (n = 5, 56%), adductors (n = 5, 31%), and hamstrings (n = 6, 9%). The seasonal distribution of injuries exhibited a trimodal pattern, with a peak at the beginning of the season (September–October), the highest peak in the middle of the season (December–January), and a last peak later in the season (March–April). The average timing of injuries during matches was at 36.3 ± 23.4 minutes, and more injuries occurred during the first half (n = 73, 71%) than the second (n = 30, 29%) (*P* < 0.01).

### Hamstring

**Figure 2.**
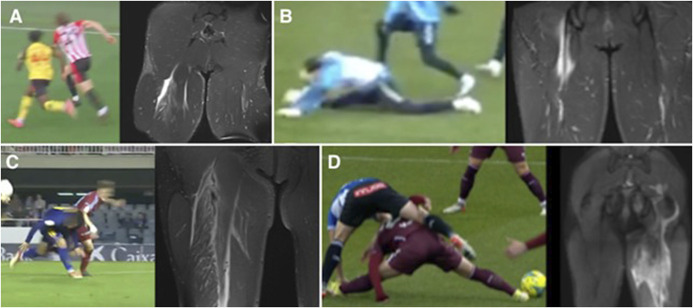
Hamstring injuries: High-speed sprinting typically affects proximal BF (A), whereas stretching (hip flexed with knee extended) usually causes proximal SM injuries (B). Mixed-type (sprinting and stretching) injuries also occur, affecting proximal BF in this example (C). The more energy, the more severe injury, and proximal BF and ST avulsion in this case (D). Hip flexion, knee extension, and trunk flexion are typically involved in hamstring injuries.

Hamstring injuries are the most common muscle-tendon injuries in football, with their proportion of all injuries doubling from 12% to 24% over 20 years.^6^ Despite this, there remains a scarcity of detailed information regarding the precise mechanisms of these injuries.^38^ Different hamstring injury mechanisms and MRI findings are presented in Figure 2A-D.

The hamstring muscle-tendon complex, comprising the biceps femoris (BF) with its long and short heads, semimembranosus (SM), and semitendinosus (ST), functions predominantly as a biarticular group that integrates hip extension and knee flexion.^39^ Distinct patterns of injury have been identified, with approximately 83%, 11%, and 5% of hamstring injuries affecting the BF, SM, and ST, respectively.^40^ The complexity and biarticular nature of these muscles contribute to varied injury mechanisms, which have been broadly categorized into two types: 'stretch-type' and 'sprinting-type.' 'Stretch-type' injuries often result from extensive hip flexion with an extended knee, whereas 'sprinting-type' injuries typically occur during eccentric contractions in the late swing phase of running.^41^ A recent systematic review highlighted these distinctions, noting that stretch-type injuries commonly affect the proximal tendons of the SM or BF, while sprinting-type injuries predominantly affect the long head of the BF.^42^ The most severe avulsions involving BF, SM, and/or ST tend to arise from rapid, forceful hip flexion with the knee extended.^43^ The knowledge of the different hamstring injury mechanisms can help in identifying the injury location and the prognosis of the injury,^42–44^ other than designing the best rehabilitation and prevention programs.^45^

The study by Gronwald et al^32^ focused on noncontact and indirect contact match hamstring injuries recorded over four seasons (2014-2019) in the top two divisions of German male football. The study included 52 cases of hamstring injuries, which were categorized into sprint-related (48%, median time loss 25 days) and stretch-related (52%, median time loss 21 days) injuries. Sprint-related injuries occurred exclusively during linear acceleration or high-speed running, while stretch-related injuries were associated with CKC movements such as braking or stopping with a lunging or landing action, and open chain movements such as kicking. The kinematic analysis of stretch-related injuries revealed a change in movement, transitioning from knee flexion to knee extension, and a knee angle of less than 45 degrees at the assumed injury frame in all observed chain movements. BF was identified as the most affected muscle, comprising 79% of all cases.

Jokela et al^26^ present a detailed exploration of acute high-grade hamstring injuries among male soccer players through systematic video analysis paired with MRI findings. Conducted between September 2017 and January 2022 across two specialized sports medicine hospitals, this research scrutinizes 14 cases of acute hamstring injuries in 13 professional male soccer players aged between 18 and 40 years (one goalkeeper, four defenders, three midfielders, and five forwards). Through video analysis, this descriptive case series study identified three main injury mechanisms: mixed-type (encompassing both sprint-related and stretch-related mechanisms at 43%), stretch-type (36%), and sprint-type (21%). The injuries predominantly occurred during high or very high horizontal speed movements (71%) and were chiefly seen in isolated proximal BF (36%). The typical body positions at the moment of injury highlighted were neutral trunk (43%), hip flexion between 45 and 90° (57%), and knee flexion less than 45° (93%). The MRI findings corroborated that 79% were isolated single-tendon injuries. This insightful analysis demonstrates that most hamstring injuries in soccer result from high-speed movement (typically involving hip flexion, knee extension, and trunk flexion), urging physicians to anticipate proximal and isolated single-tendon injuries, predominantly in the BF, if such mechanisms are observed during gameplay.

Adding to the comprehensive understanding of hamstring injuries, the recent study by Della Villa et al^34^ compared the mechanisms of hamstring injuries in professional male (Italian Serie A) and female (both in club and national team) football players. As the professionalism of female elite football has evolved, players are exposed to higher physical demands, increasing the risk of muscle injuries. This study, conducted on 129 severe injuries, highlighted notable gender differences in injury contexts and mechanisms. Three in every five injuries (62%) in women and two in every five for men (39%) occurred during running. Overstretching-type injuries collectively accounted for almost half of injuries (46%) in men but only one-fifth in women (20%). Kicking injuries were also common, accounting for one out of six injuries for women (17%), and one of 10 in men (10%). Remarkably, noncontact injuries were more prevalent among female players, suggesting differences in muscle utilization and movement patterns between sexes. The same four situational patterns accounted for nearly all injuries in both sexes, including running, kicking, and overstretching during open and CKC movements. Women players experienced more running and kicking injuries, as well as fewer overstretching injuries, than men, particularly during lower-speed maneuvers. This discrepancy may be attributed to differences in tactical roles or physical conditioning.

Vermeulen et al^37^ challenged the traditional assumption that hamstring injuries primarily occur at top speeds. Their video analysis of 63 sudden-onset hamstring injuries in the Qatar Stars League (2013-2020) revealed that 86% of injuries involved running, but many did not occur at maximal speed. Acceleration over short distances (<10 m) was the most common injury mechanism (24%), while pressing situations were involved in 46% of injuries, often with indirect contact and inadequate balance playing significant roles. They found that indirect player-to-player contact and sudden decelerations contributed to 53% of injuries occurring at short distances (0-10 m), while pressing actions increased injury risk, with a higher incidence of contact when the injured player was pressing an opponent (69%) compared with when they were being pressed (15%). Their study highlights the importance of considering factors beyond high-speed running as a primary risk factor for hamstring injuries. Instead, unexpected short accelerations, indirect contact, and situational game dynamics are critical in injury causation.

### Adductors

Adductors injuries constitute 23% of muscle-tendon injuries in football, with groin injuries ranking among the most common afflictions.^8^ A 25-player squad can anticipate experiencing two to four acute groin injuries each season. Remarkably, the hip adductor muscles are implicated in two-thirds of acute groin injuries among athletes, with the adductor longus being the most frequently injured, accounting for 90% of these cases^46^ Consequently, the adductor longus emerges as a critical focus for the prevention of acute groin injuries despite the current lack of comprehensive understanding of the mechanisms behind these injuries.

The paper by Serner et al^33^ provides a comprehensive investigation into the circumstances leading to acute adductor longus injuries among 17 male professional football players (27.5 mean age): there were four goalkeepers, four defenders, eight midfielders, and one forward (15 players from the highest Qatar national league and two in the second highest league). Using a cross-sectional study design, the researchers conducted a detailed visual analysis of video footage to describe and categorize the inciting events associated with these injuries. The study included players who had suffered acute adductor longus injuries. The researchers found that most injuries occurred in noncontact situations (71%) and were often the result of a quick reaction to a change in play (53%). The actions leading to injury were identified as change of direction (35%), kicking (29%), reaching (24%), and jumping (12%). The study further differentiated the actions into CKC and OKC, revealing that change of direction and reaching were predominantly closed chain movements, characterized by hip extension and abduction with external rotation. By contrast, kicking and jumping were classified as open chain movements, involving a shift from hip extension to hip flexion, and from hip abduction to adduction, with the hip externally rotated. The findings have significant implications for developing prevention strategies, suggesting that training aimed at increasing the muscle's capacity to withstand rapid activation at a lengthened state might effectively reduce injury risk.

**Figure 3.**
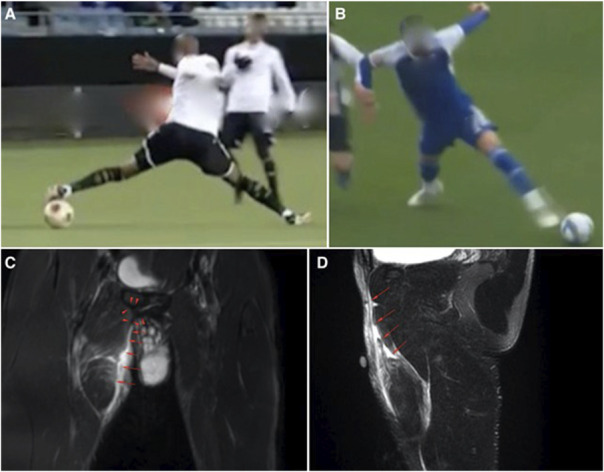
Adductor injuries: Severe adductors injuries often occur during closed kinetic chain stretching movements, such as reaching for ball with the uninjured leg (A, B). These injuries are typically characterized by hip extension, abduction, and external rotation. Examples of the possible MRI findings showing acute proximal adductor longus tear are presented (C, D). Among other possible adductor injury mechanisms are open kinetic chain stretching (pass kick, reaching with injured leg etc.) and rapid movements, such as sprinting, dribbling, and changing direction. The mechanisms are often more complicated in adductor injuries.

Building upon this foundation, Jokela et al^35^ conducted a systematic visual video analysis to explore severe acute adductor longus injuries in 20 professional male soccer players (median age: 27 years, range 18-35 years). This cross-sectional study focused exclusively on high-grade injuries, either complete adductor longus tendon ruptures or severe partial lesions, all resulting in an absence of >28 days. Regarding injury mechanisms, they found that 65% of injuries were noncontact, while 35% involved indirect contact. Most (70%) of injuries occurred in CKC actions, primarily when the player was reaching with the uninjured leg, while only 15% of injuries occurred during high-speed running, suggesting that not all adductor injuries are sprint-related. Furthermore, reaching with the injured leg (OKC stretching) accounted for 10% of cases. Key biomechanical findings were stated, including CKC injuries were consistently characterized by hip extension, hip abduction, and external rotation (found in 64% of cases), OKC injuries featured hip abduction, external rotation, and a rapid shift from hip extension to flexion. Moreover, eccentric muscle activation played a key role, particularly during sudden changes in movement or excessive loading. The study emphasizes that not all adductor injuries result from high-speed actions, and mechanical load during directional changes, reaching, and closed-chain actions play a substantial role in injury causation. Typical CKC stretching injury mechanisms and MRI findings are presented in Figure 3A-D.

### Rectus Femoris

**Figure 4.**
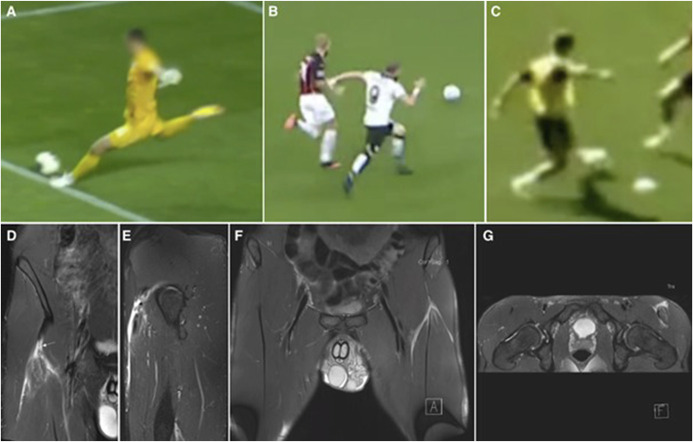
Rectus femoris injuries: Kicking is the most common mechanism (A), but the injuries can also occur during rapid movements requiring eccentric muscle action, such as sprinting (B) and change of direction (C). Complete ruptures (D, E) are typically caused by kicking or sprinting, whereas change of direction injuries cause mainly partial ruptures (F, G).

Rectus femoris (RF) injuries are notably prevalent among football players, with quadriceps muscle-tendon injuries being common in sports that involve extensive sprinting and kicking.^4,47^ When deciding the best treatment for a muscle-tendon injury, a correct diagnosis and injury classification are the first steps.^48^ The primary mechanisms underlying RF injuries are multifactorial, with kicking and sprinting identified as the predominant actions leading to such injuries.^49,50^ Specifically, kicking significantly contributes to complete tears and injuries occurring at the proximal free tendon, underlining the need for a precise understanding of injury mechanisms. Examples of kicking, sprinting, and change of direction injury mechanisms and typical MRI findings are presented in Figure 4A-G.

Jokela et al^30^ thoroughly examined the mechanisms and MRI characteristics of acute RF injuries in soccer players. Conducted from November 2017 to July 2022, this descriptive case series analyzed injuries across two specialized sports medicine hospitals involving 20 injuries in 19 professional male soccer players aged 18 to 40 years (4 goalkeepers and 15 outfield players). The inclusion criteria required a confirmed RF injury on MRI within 7 days post-injury and the availability of video footage of the incident. The study identified three main injury mechanisms: kicking (80%), sprinting (10%), and changing direction (10%). It was found that isolated single-tendon injuries comprised 60% of the cases. Among kicking injuries, 62.5% included complete tendon ruptures. The study also highlighted the involvement of the direct tendon in 33% of isolated injuries and the common tendon in all combined injuries.

### Methodological Appraisal

Table 2 summarizes the methodological evaluation of the 10 included studies. Common strengths identified across the included studies include clear objectives, thorough method descriptions, and insightful integration of findings within the broader context of existing literature. However, areas highlighted for improvement—such as the need for detailed reporting of sample characteristics, enhanced clarity on video source quality, and the inclusion of control groups—suggest pathways to bolster future research endeavors. Particularly, the studies included in our review varied in their systematic approach to video analysis, depth of reporting results, and elucidation of injury contexts, indicating an overarching need for methodological refinement to advance the precision and applicability of findings in sports injury prevention and treatment strategies.

**TABLE 2.** Methodological Appraisal of the Included Studies

QA-SIVAS Item	Della Villa et al^50^	Della Villa et al^49^	Gronwald et al^20^	Jokela et al^25^ (Hamstring)	Jokela et al^24^ (Rectus Femoris)	Klein et al^28^	Serner et al^44^	Aiello et al^36^	Jokela et al^35^ (Adductors)	Vermeulen et al^37^
1. Clear statement of objectives	Yes	Yes	Yes	Yes	Yes	Yes	Yes	Yes	Yes	Yes
2. Justification of study design	Yes	Yes	Yes	Yes	Yes	Yes	Yes	Yes	Yes	Yes
3. Detailed description of sample characteristics	Yes	Yes	Yes	Yes	Yes	Yes	Yes	Yes	Yes	Yes
4. Detailed description of video source and quality	No	Yes	Yes	Yes	No	Yes	Yes	No	Yes	Yes
5. Detailed description of methodology	Yes	Yes	Yes	Yes	Yes	Yes	Yes	Yes	Yes	Yes
6. Systematic approach to video analysis	Yes	Yes	Yes	Yes	Yes	Yes	Yes	Yes	Yes	Yes
7. Inclusion of relevant data sources	Yes	Yes	Yes	Yes	Yes	Yes	Yes	Yes	Yes	Yes
8. Description of rater expertise	Yes	Yes	No	No	No	Yes	No	No	Yes	Yes
9. Findings are observed by more than one researcher	Yes	Yes	Yes	Yes	Yes	Yes	Yes	Yes	Yes	Yes
10. Inclusion of a control group	No	No	No	No	No	No	No	No	No	No
11. Use of validated methods	Yes	Yes	Yes	Yes	Yes	Yes	Yes	Yes	Yes	Yes
12. Detailed reporting of main results	Yes	Yes	Yes	Yes	Yes	Yes	Yes	Yes	Yes	Yes
13. Report of absolute numbers or proportions of injury case	Yes	Yes	Yes	Yes	Yes	Yes	Yes	Yes	Yes	Yes
14. Details about the injury context	Yes	Yes	Yes	Yes	Yes	Yes	Yes		Yes	Yes
15. Inclusion of example screenshots/video frames	Yes	Yes	Yes	Yes	Yes	Yes	Yes	No	Yes	Yes
16. Findings are discussed	Yes	Yes	Yes	Yes	Yes	Yes	Yes	Yes	Yes	Yes
17. Clinical/practical implications of the results are discussed	Yes	Yes	Yes	Yes	Yes	Yes	Yes	Yes	Yes	Yes
18. Limitations are addressed	Yes	Yes	Yes	Yes	Yes	Yes	Yes	No	Yes	Yes

## DISCUSSION

Understanding the mechanisms behind muscle-tendon injuries is crucial for developing effective prevention and treatment strategies, particularly with the aid of video analysis. Our review included 10 studies investigating three main types of thigh injuries, revealing a predominant occurrence of severe thigh muscle-tendon injuries from noncontact events (Table 3).

**TABLE 3.** Focus on Video Analysis Studies About Muscle-Tendon Injuries in Football Players

Severe muscle injuries are mostly noncontact
Eccentric muscle action is the key
Hamstring: Sprinting (acceleration/deceleration) Rectus femoris: kicking, sprinting Adductor: Complicated, open/closed chain

### Hamstrings

The studies of Della Villa et al^29^ and Gronwald et al^32^ emphasized the prevalence of acceleration injuries, particularly noting that such incidents account for a significant proportion of hamstring injuries. This is attributed to the large forces, negative muscle work, and high activation of the hamstring muscles, specifically the BF during the late swing phase, underscoring its high-risk nature as an inciting event.^51^ Indeed, rupture occurs typically when there is an abrupt acceleration or deceleration. Aiello et al^36^ further supported this, demonstrating that 88% of hamstring injuries occurred when players ran at speeds exceeding 25 km/h, often at more than 80% of their maximal sprinting velocity. These findings suggest that reducing exposure to high-speed running, as sometimes recommended, may not be a suitable prevention strategy, as hamstring injuries often occur at peak running speeds. Instead, preparing players for such intensities through targeted training may be more effective. Differently, Vermeulen et al^37^ revealed that 86% of injuries involved running, but many did not occur at maximal speed. Acceleration over short distances (<10 m) was the most common injury mechanism (24%), while pressing situations were involved in 46% of injuries, often with indirect contact and inadequate balance playing significant roles. This contrasts with Aiello et al,^36^ reinforcing the need to differentiate between high-speed running and short, explosive accelerations as risk factors.

Building on these insights, a recent study by Della Villa et al.^34^ her illuminates the specific injury mechanisms in male and female football players, revealing significant gender differences. The study highlights that while running-related injuries are common to both, women tend to have a higher proportion of noncontact injuries during these activities compared with men. This distinction highlights potential variations in biomechanics and training, such as differences in fatigue patterns and neuromuscular control, which may influence susceptibility to various types of injuries. The high prevalence of noncontact injuries in female athletes, especially during running and kicking, underscores the need for tailored preventive measures that consider these biomechanical and physiological differences. Beyond biomechanical factors, differences in gameplay intensity, movement strategies, and muscle activation (possibly related to the interaction between menstrual cycle phases and ligament laxity) may also contribute to higher injury rates in female athletes, particularly during running and kicking.^52^ Studies have suggested that lower limb alignment, decreased hamstring-to-quadriceps strength ratio, and landing mechanics could predispose female athletes to a greater risk of noncontact muscle injuries. This could lead to more effective gender-specific interventions to reduce the incidence of hamstring and other muscle-tendon injuries in sports.

### Adductors

Contrasting findings between the studies of Della Villa et al^29^ and Serner et al^33^ regarding running-based adductor injuries highlight the potential variability in injury mechanisms for adductor longus strains, possibly influenced by the severity of injuries studied. Aiello et al^36^ identified three adductor injuries, each occurring in distinct game situations: one during deceleration, one during acceleration while controlling the ball, and one while performing an instep kick. These results align with Serner et al,^33^ who identified change of direction and kicking as common mechanisms for adductor injuries.

Building upon this, Jokela et al^35^ revealed that 70% of injuries occurred in CKC actions, primarily during reaching with the uninjured leg. These findings reinforce the role of rapid eccentric loading in adductor injuries, with movements characterized by hip extension, hip abduction, and external rotation being particularly risky. Unlike Serner et al,^33^ who found a higher proportion of change-of-direction injuries, Jokela et al^35^ highlighted that most injuries occurred in CKC situations rather than OKC scenarios, suggesting that adductor injuries may be more commonly linked to stabilization and sudden eccentric loading than previously believed.

### Rectus Femoris

This is in line with the study by Della Villa et al,^29^ kicking injuries emerged as a dominant pattern for quadriceps injuries, corroborating earlier research.^49^ Kicking was also a typical pattern for adductor injuries in the cohort of Della Villa et al,^29^ with a nearly identical proportion to the work of Serner et al^33^ on football adductor longus injuries (31% vs 29%). The kicking is associated with a high activation rate, stretch, and muscle elongation during the backswing phase, potentially leading to overstretching or rapid eccentric actions necessary for decelerating the limb.^53^ Interestingly, hamstring activation is comparatively low during the initial phases of ball kicking, highlighting a distinct pattern of muscle engagement.^53^

### Limitations

Despite the valuable insights gained from this review, several limitations should be acknowledged. First, our review is based on Level 3 evidence according to the Oxford Centre for Evidence-Based Medicine, meaning that conclusions are drawn from non-randomized observational studies, which limits the ability to establish causation. Second, there is methodological variability among the included studies, as assessed using the QA-SIVAS scale. Differences in video analysis approaches, rater expertise, and video quality may affect the consistency and reliability of findings (Table 2). Third, our findings are specific to professional football settings, and caution is needed when generalizing these results to amateur or youth levels, where biomechanical and physical differences may alter injury risk. Fourth, although we expanded our review to include 10 studies, the number of available studies remains relatively small, which limits the ability to draw broader conclusions. Fifth, the recommendations are categorized as SORT level B, indicating that while video analysis provides useful observational data, the supporting evidence remains limited due to inconsistencies in methodology and study design. In addition, our reliance on peer-reviewed journal articles may introduce publication bias, as studies with significant findings are more likely to be published. Finally, the practical application of video analysis for injury prevention in real-world settings faces challenges, including financial costs, the need for specialized training, and data storage concerns. Furthermore, while our review primarily focused on noncontact and indirect contact injuries, it is also important to acknowledge that direct contact injuries significantly contribute to muscle-tendon injuries in football. Studies focusing on muscle strains often exclude direct trauma-related injuries, which can result from tackles, collisions, or falls. Future studies should incorporate video analysis techniques to better understand the biomechanics and risk factors associated with direct contact muscle injuries. Addressing these limitations in future research will enhance the applicability and robustness of video analysis in football injury prevention.

### Practical Applications

The increased understanding of injury mechanisms gleaned from video analysis offers valuable insights, helping to plan targeted interventions designed to reduce the prevalence of these injuries in sports (Table 4).

**TABLE 4.** Practical Applications of Video Analysis in Muscle Injuries

Detailed observation	It allows for frame-by-frame examination of the injury event, capturing the exact movements and positions of the players involved. This detailed observation helps identify the specific actions and biomechanical factors that contribute to the injury
Identification of (more) patterns	By analyzing multiple injury incidents, it can reveal common patterns and scenarios that lead to injuries, other than occurring during sprinting, kicking, or sudden changes in direction
Contextual understanding	It may provide context to the injury, such as the player's interaction with the ball, other players, and the overall game situation. This helps in understanding the situational factors that may increase injury risk
Noncontact vs Contact Injuries	Not only noncontact injuries but also analyzing contact injuries may help to better understand of how different types of injuries occur
Biomechanical insights	It can highlight the role of biomechanical factors, such as joint angles, muscle loading, speed of limbs, and body posture, in injury mechanisms
Validation of theories	Providing empirical evidence to support or refute existing theories about injury mechanisms is important
Development of prevention strategies	By understanding the specific mechanisms and contexts of injuries, it informs the development of targeted injury prevention strategies. Medical staff can use this information to modify training practices and reduce injury risk
Rehabilitation and return-to-play	Insights from video analysis can also guide rehabilitation protocols by identifying the movements that need to be addressed during recovery

### Future Directions

The potential role of AI, mainly through the implementation of deep learning techniques as demonstrated in a recent study^54^ has a vast potential to transform video analysis:

1. Automated Event Detection: AI can automatically detect and classify events in sports videos, such as specific movements or actions associated with a higher risk of injury. This automation significantly reduces the time and effort required for manual video analysis.
2. Pattern Recognition: AI algorithms can identify complex patterns and correlations in large data sets that may not be immediately apparent to human analysts. This helps in uncovering subtle risk factors and injury mechanisms.
3. Real-Time Analysis: AI can process video footage in real-time, providing immediate feedback to athletes and coaches. This enables on-the-spot adjustments to techniques and training practices, helping to prevent injuries.
4. Enhanced Accuracy: AI models, particularly those using deep learning techniques, can achieve high levels of accuracy in identifying and analyzing movements. This precision helps in better understanding the biomechanics of injury-prone actions.
5. 3D Motion Analysis: AI can create three-dimensional models of athletes' movements from video footage. This 3D analysis offers a more comprehensive understanding of the biomechanics involved, facilitating more effective injury prevention strategies.
6. Data Integration: AI can integrate data from multiple sources, such as video footage, wearable sensors, and medical records, to provide a holistic view of an athlete's condition and risk factors. This integrated approach enhances the ability to predict and prevent injuries.
7. Predictive Analytics: AI can analyze historical data to predict future injury risks. AI can help develop proactive measures to prevent similar injuries by identifying patterns and trends in past injuries.
8. Customized Training Programs: AI can tailor training programs to meet the individual needs and vulnerabilities of each athlete. By analyzing an athlete's unique movement patterns and risk factors, AI can recommend personalized exercises and techniques to reduce injury risk.
9. Continuous Learning: AI systems can continuously learn and improve from new data. As more video footage and injury data are analyzed, AI models become more accurate and effective in identifying risk factors and suggesting preventive measures.
10. Resource Efficiency: By automating the analysis process, AI frees up valuable time for coaches and medical staff, allowing them to focus more on implementing preventive strategies and providing personalized care to athletes.

The potential application of AI in video analysis studies enhances the quality and depth of analytical outputs and opens new avenues for research and development in sports science and analytics. Future research should focus on validating AI-based injury detection models, optimizing real-time video processing, and expanding AI-driven injury prevention frameworks across different sports and competition levels.

## CONCLUSIONS

This study comprehensively analyzes thigh muscle-tendon injuries in football athletes, leveraging video analysis to unveil detailed mechanisms and situational patterns. By systematically reviewing and categorizing the types of injuries observed, this research not only enriches our understanding of how these injuries occur but also highlights the predominant risk factors associated with various football activities, such as sprinting, kicking, and direction changes. The sports medicine community must continue to refine these methodologies and explore innovative technologies, such as AI, to enhance injury prevention and treatment efforts.
